# Pharmacodynamic effects of the K^+^ binder patiromer in a novel chronic hyperkalemia model in spontaneously hypertensive rats

**DOI:** 10.14814/phy2.14572

**Published:** 2020-09-23

**Authors:** Sai Prasad N. Iyer, Lawrence Lee, Lingyun Li

**Affiliations:** ^1^ Medical and Scientific Affairs Relypsa, Inc., a Vifor Pharma Group Company Redwood City CA USA

**Keywords:** aldosterone, chronic kidney disease, hyperkalemia, patiromer

## Abstract

Currently described hyperkalemia (HK) animal models are typically acute and cause significant distress and mortality to the animals, warranting new approaches for studying chronic HK in a more appropriate clinical setting. Using the spontaneously hypertensive rat (SHR) model as a more relevant disease template, as well as surgical (unilateral nephrectomy), dietary (3% potassium [K^+^] supplementation), and pharmacological (amiloride) interventions, we were able to stably induce HK on a chronic basis for up to 12 weeks to serum K^+^ elevations between 8 and 9 mmol/L, with minimal clinical stress to the animals. Short‐term proof‐of‐concept and long‐term chronic studies in hyperkalemic SHRs showed concomitant increases in serum aldosterone, consistent with the previously reported relationship between serum K^+^ and aldosterone. Treatment with the K^+^ binder patiromer demonstrated that the disease model was responsive to pharmacological intervention, with significant abrogation in serum K^+^, as well as serum aldosterone to levels near baseline, and this was consistent in both short‐term and long‐term 12‐week chronic studies. Our results demonstrate the feasibility of establishing a chronic HK disease state, and this novel HK animal model may be suitable for further evaluating the effects of long‐term, K^+^‐lowering therapies on effects such as renal fibrosis and end‐organ damage.

## Graphical abstract

Currently described hyperkalemia (HK) animal models are typically suboptimal for studying chronically elevated potassium, warranting new approaches for studying chronic HK. Using the spontaneously hypertensive rat model as well as surgical and minimal drug and dietary interventions, we achieved a reproducible animal disease model that maintains a stable, chronic HK state. Our results showed that the potassium binder, patiromer significantly reduced serum potassium and aldosterone in hyperkalemic rats, which may be suitable for further evaluating potassium‐lowering therapies, as well as evaluating long‐term effects on end‐organ damage and in studies of aldosterone regulation and actions.

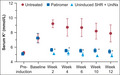

## 1 INTRODUCTION

Excess serum potassium (K^+^), or hyperkalemia (HK), a potentially life‐threatening condition, is prevalent in many patients with stage 3 or greater chronic kidney disease (CKD). Hyperkalemia is particularly common in CKD patients with concomitant diabetes mellitus (DM) and/or heart failure (HF) and who are on renin‐angiotensin‐aldosterone system inhibitors (RAASi) for treatment of their underlying disease (Albert et al., 2009; Einhorn et al., 2009; Palmer, 2004; Yildirim et al., 2012). Evidence from observational studies has shown that risk of chronic and recurrent HK is high in these patients (Einhorn et al., 2009; Wang et al., 2013), warranting the need for a chronic management approach, as well as the availability of suitable animal disease models that more accurately reflect the clinical pathological condition.

Very few scientific studies describe experimental methods of inducing HK in rodents, specifically rats. Most studies primarily focus on inducing CKD with the resulting HK being a transient effect in these models, often using a subtotal nephrectomy (Nx), which is more commonly referred to as a 5/6 Nx (Baba, 1986). In this model, HK is typically acute and may require additional dietary, pharmaceutical, or chemical intervention to induce and prolong the condition (Borok, Schneider, Fraley, & Adler, 1987; Diwan, Brown, & Gobe, 2018; Diwan, Mistry, Gobe, & Brown, 2013; Layton, Edwards, & Vallon, 2018; Li et al., 2016; Ma, Zhang, Guo, & Xu, 2014; Wapstra, van Goor, de Jong, Navis, & de Zeeuw, 1999). These interventions are sufficient to induce HK (Li et al., 2016), but the duration is often short or clinical signs and symptoms of distress in these animals may be severe enough to warrant early termination of the study (Diwan et al., 2013, 2018; Li et al., 2016; Wapstra et al., 1999), often with high mortality (Askari, Seifi, & Kadkhodaee, 2016). These conditions are not feasible for evaluating chronic HK treatments in a preclinical setting.

Patiromer is a sodium‐free, nonabsorbed K^+^‐binding polymer approved for the treatment of HK in adults (Veltassa, 2015). Patiromer has demonstrated efficacy and was shown to be generally well tolerated when administered chronically across multiple clinical trials in the treatment of HK in patients with CKD, DM, and/or HF (Bakris et al., 2015; Pergola et al., 2017; Weir et al., 2015). Patiromer was also evaluated in the treatment of HK in patients on hemodialysis (Bushinsky et al., 2016), as well as in the prevention of HK in patients with HF treated with spironolactone (Pitt et al., 2011, 2018) and resistant hypertension (HTN) and advanced CKD (Agarwal et al., 2019).

The goals of the current study were to develop a novel chronic HK animal model using dietary, pharmacological, and surgical interventions in the context of an appropriate background pathological template, such as the spontaneously hypertensive rat (SHR) (Okamoto & Aoki, 1963), and to evaluate the effect of patiromer on serum K^+^ and aldosterone levels in the model.

## 2 MATERIALS AND METHODS

### 2.1 Establishment of chronic HK in SHRs

Male SHRs, 7–9 weeks old, weighing 200–250 g with unilateral nephrectomies (UniNx) were brought in‐house and acclimated initially on standard rodent chow (e.g., LabDiet Rodent 5001, Lab Supply, Fort Worth, TX) for 2–3 days and then for 2 weeks on a purified and refined, low‐divalent cation diet (Teklad Diet [TD] 04498, Envigo, Indianapolis, IN) and deionized water ad libitum. A blood collection was performed to ensure that serum K^+^ levels were still in the normal range (3.5–5.5 mmol/L). Blood was collected in yellow‐capped SAI Technologies serum collection tubes and allowed to coagulate, then placed on ice. Tubes were centrifuged to obtain serum and stored on ice prior to analysis. Samples were visually examined to screen for hemolysis. The animals were switched to another purified, refined diet (TD160304 and TD160305, Envigo, Indianapolis, IN) supplemented with additional K^+^ (2% and 3% weight/weight, respectively) only and in combination with amiloride (0.025–0.05 mM), a K^+^‐sparing diuretic, given through drinking water. The animals remained on this induction period for 5–13 days, at which point blood was collected to confirm induction of HK by analyzing serum K^+^ levels (mean K^+^ >6.5 mmol/L), making this a baseline reading.

All animal experiments were conducted in accordance with the Guide for the Care and Use of Laboratory Animals from the Institute for Laboratory Animal Research of the National Research Council. All study protocols were approved by LifeSource Biomedical's Institutional Animal Care and Use Committee prior to the initiation of the studies.

### 2.2 Short‐term proof‐of‐concept, dose response, and chronic treatment of SHR‐HK with patiromer

For short‐term proof‐of‐concept (POC) treatment of hyperkalemic SHRs (SHR‐HK), the animals were randomized into an untreated group, where they remained under induction conditions, or into a treatment group, where the animals were also under induction conditions, but were treated starting on day 5 with 4 g kg^‐1^ day^‐1^ of patiromer (via oral gavage) for 8 days. The dosing volume for the oral gavage was 3 ml and solutions contained 0.11% xanthan gum. Untreated animals were administered a vehicle control. For the patiromer dose‐response study, SHR‐HK were randomized into untreated, 0.5, 1.5, and 4.5 g kg^‐1^ day^‐1^ patiromer groups and treated for 11 days from the baseline time point. Patiromer was mixed in the chow in this study. Control animals included in the study were uninduced SHRs with UniNx and wild‐type Wistar Kyoto (WKY) rats. In the chronic‐treatment study, SHR‐HK were randomized into the untreated or patiromer group (4 g kg^‐1^ day^‐1^) and treated for 12 weeks. During and at the end of all treatment periods, blood was collected to measure serum K^+^ and aldosterone levels in all groups. Serum K^+^ was measured using the ACE Axcel Chemistry Analyzer (Alfa Wassermann Diagnostic Technologies LLC, West Caldwell, NJ).

### 2.3 Measurement of serum aldosterone

Serum aldosterone was measured using an ALPCO Aldosterone ELISA kit (Salem, NH). Frozen samples were thawed and assayed for aldosterone levels at various dilutions to ensure that measurements were within the assay‐kit range. Final levels were calculated by generating a standard curve from aldosterone standards provided with the kit.

### 2.4 Statistical analysis

Assay results were analyzed for statistical significance using either two‐way ANOVA followed by post hoc Bonferroni's multiple comparison posttest using Prism v8.1.2 (GraphPad, San Diego, CA) or using the student's *t* test (two‐sample, assuming equal variances) with Excel. Regression analysis for the correlation of serum K^+^ and aldosterone was also performed using Prism v8.1.2.

## 3 RESULTS

### 3.1 HK induction in SHRs — effect on serum K^+^ and aldosterone

**FIGURE 1:**
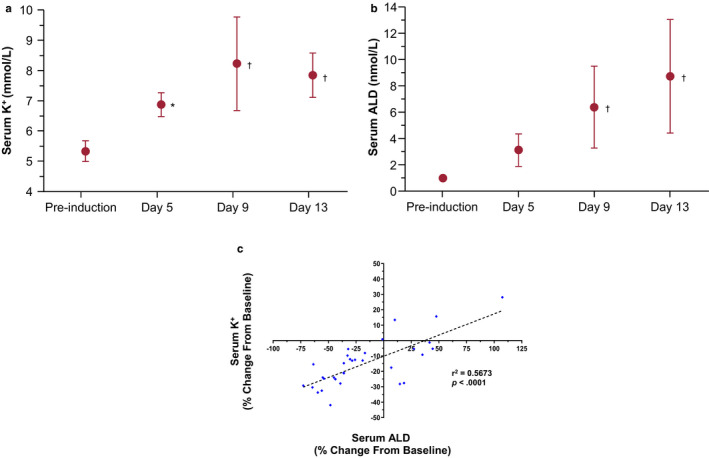
Serum K^+^ and aldosterone (ALD) increase upon hyperkalemia (HK) induction in spontaneously hypertensive rats (SHRs). SHRs were subject to UniNx followed by induction with a high K^+^ diet (3%) and treatment with 0.05 mM amiloride. (a) Serum K^+^ is markedly elevated in SHRs from pre‐induction through day 13 in a novel induction approach. Data shown as mean ± standard deviation (*SD*; *N* = 9). (b) Effect on serum ALD upon HK induction from pre‐induction through day 13. Data shown as mean ± *SD* (*N* = 9). (c) Correlation of the change from baseline between serum K^+^ and ALD per linear regression analysis. Two‐way ANOVA followed by post hoc Bonferroni's multiple comparison posttest was used to compare time points versus pre‐induction: *p < .05; ^†^ *p* < .0001

Initial studies using UniNx and dietary K^+^ supplementation (2%–3%) did not induce elevated serum K^+^ in SHRs (5.7–6.0 mmol/L at day 14 vs. 6.1 mmol/L at pre‐induction, *n* = 6). Therefore, the K^+^‐sparing diuretic amiloride was added to the treatment regimen to induce HK in the subsequent studies. UniNx, dietary K^+^ supplementation (3%), and amiloride treatment of SHRs (*N* = 9) resulted in significantly elevated serum K^+^ levels after 5 days (5.3 mmol/L [pre‐induction] vs. 6.8 mmol/L, *p* = .02), and animals remained hyperkalemic over a total of 2 weeks, reaching a peak of ~8.2 mmol/L at day 9 (*p* < .0001) and 7.8 mmol/L at day 13 (*p* < .0001) (Figure 1a). Serum aldosterone levels were also concomitantly significantly increased (3.2, 6.4, and 8.8 nmol/L at days 5, 9, and 13, respectively; *p* < .0001 at days 9 and 13 vs. pre‐induction, 1.1 nmol/L; Figure 1b). Regression analysis indicated that changes from baseline in serum levels of aldosterone and K^+^ were correlated (*r* ^2^ = .57, *p* < .0001) (Figure 1c). Despite the elevated levels of serum K^+^ and minor weight loss (when compared with baseline) in some animals (Table 1a), no other clinical signs of distress were observed, indicating that the SHRs were able to tolerate the induction process, allowing for establishment of a chronic HK disease animal model.

**TABLE 1:** Weight data from (a) model‐establishment, (b) dose‐response, and (c) long‐term studies. *SD*, standard deviation

(a) Animal number	Preinduction	Day 5	Day 13
1	270.2	311.5	310.2
2	273.3	328.9	334.3
3	274.1	300.2	307.8
4	292.2	294.8	297.5
5	294.8	273.3	266.1
6	281.8	274.1	276.4
Mean weight (g)	281.1	297.1	298.7
*SD*	10.4	21.6	24.7
% change		5.7	6.3

(b) Group	Preinduction	Baseline			Day 4			Day 8		
	Mean weight (g)	Mean weight (g)	Mean weight (% change)	*SD*	Mean weight (g)	Mean weight (% change)	*SD*	Mean weight (g)	Mean weight (% change)	*SD*
Untreated	298.3	288.1	−3.4	1.9	287.3	−3.7	2.3	290.1	−2.7	1.3
0.5 g/kg patiromer	303.2	292.7	−3.5	2.0	289.7	−4.5	2.8	295.3	−2.6	2.9
1.5 g/kg patiromer	305.4	293.3	−3.9	1.2	298.0	−2.4	1.8	302.3	−1.0	1.9
4.5 g/kg patiromer	308.7	297.5	−3.6	0.6	304.4	−1.4	1.1	306.0	−0.9	0.5
Uninduced SHR + UniNx	327.3	333.0	1.7	1.2	338.3	3.4	1.0	342.3	4.6	1.4
WKY	297.6	303.3	1.9	1.0	306.4	2.9	4.6	318.6	6.6	0.9

(c) Group	Baseline		Week 1		Week 3		Week 4		Week 5		Week 7		Week 8		Week 9		Week 11		Week 12	
	Mean weight (g)	*SD*	Mean weight (g)	*SD*	Mean weight (g)	*SD*	Mean weight (g)	*SD*	Mean weight (g)	*SD*	Mean weight (g)	*SD*	Mean weight (g)	*SD*	Mean weight (g)	*SD*	Mean weight (g)	*SD*	Mean weight (g)	*SD*
Untreated	276.9	10.8	275.1	10.9	298.7	12.4	298.5	14.2	314.3	16.5	332.4	19.2	340.7	18.9	343.3	17.7	352.0	22.8	355.0	23.0
4 g/kg patiromer	279.1	9.7	280.9	8.8	309.6	8.0	315.1	8.2	328.7	8.7	343.5	10.0	348.1	11.6	354.5	9.9	369.0	12.1	370.0	12.0
Uninduced SHR + UniNx	295.7	8.1	301.8	9.9	332.7	11.4	344.9	10.4	343.7	23.8	369.4	12.4	350.8	13.5	379.8	13.8	389.8	12.4	—^*^	—^*^

Note

Percent change is from pre‐induction.

^*^ Data are not available.

### 3.2 Further characterization of SHR‐HK model— responsiveness to a K^+^ binder

**FIGURE 2:**
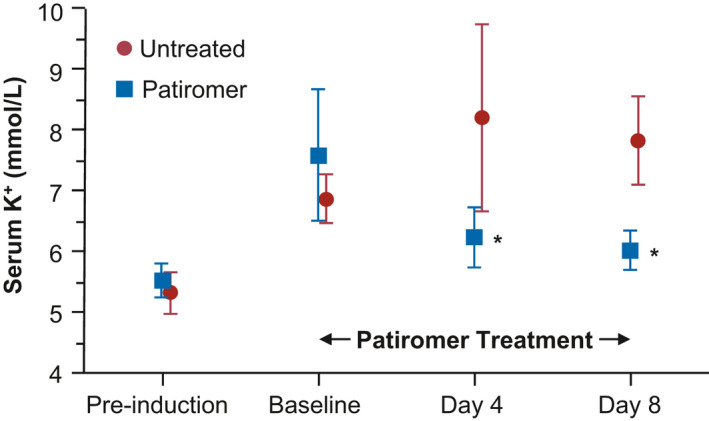
Patiromer effect in the spontaneously hypertensive rats with hyperkalemia (SHR‐HK) model (short‐term proof‐of‐concept treatment study). SHRs were induced with HK and were either untreated (red circles) or treated with 4 g kg^‐1^ day^‐1^ patiromer (blue squares). Two‐way ANOVA followed by post hoc Bonferroni's multiple comparison posttest was used to compare untreated versus treated. *p < .001

To further characterize the induced HK in the SHR model, animals were either untreated or subjected to treatment with the K^+^ binder patiromer in a short POC study. As mentioned above, patiromer has previously been shown to effectively reduce serum K^+^ levels in a HK animal model in 5/6 Nx Sprague–Dawley rats that were induced with doxorubicin, trimethoprim, and quinapril (Li et al., 2016). In this current POC study, following induction and prior to drug treatment, mean serum K^+^ levels increased from pre‐induction levels of ~5.5 mmol/L to ~6.9 mmol/L in the untreated group and to ~7.5 mmol/L in the patiromer treatment group at day 5, indicating that the induction was successful (Figure 2). This time point was designated as the baseline, and the animals were either untreated or treated with 4 g kg^‐1^ day^‐1^ of patiromer for the next 8 days. As seen in Figure 2, mean serum K^+^ levels in the treated animals were significantly reduced from baseline levels at days 4 and 8 of treatment, whereas levels in the untreated group remained high (*p* < .001 vs. untreated at both time points). This indicated that the SHR‐HK were responsive to treatment with a K^+^ binder patiromer in this short‐term POC.

### 3.3 Dose response of patiromer treatment and effect on serum K^+^

**FIGURE 3:**
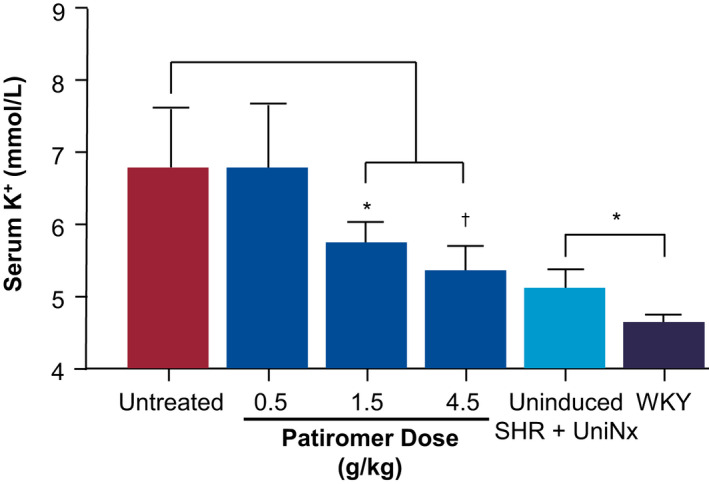
Dose‐response effect of patiromer treatment on serum K^+^. Serum K^+^ was assessed at day 11. Hyperkalemic spontaneously hypertensive rats (SHR) were either untreated or treated with increasing doses of patiromer (0.5, 1.5, 4.5 g kg^‐1^ day^‐1^) for 11 days. Control animals were uninduced SHR + UniNx and wild‐type Wistar Kyoto (WKY) rats. *N* = 8/group; data shown as mean ± standard deviation. Two‐way ANOVA followed by post hoc Bonferroni's multiple comparison posttest was used to compare untreated versus treated. The student's *t* test was used to compare uninduced SHR + UniNx versus WKY. *p < .01; ^†^ *p* < .0001

Next, we characterized the responsiveness of serum K^+^ in SHR‐HK to treatment with patiromer in a dose‐response study. HK‐induced SHRs were either untreated or treated with increasing amounts of patiromer (0.5, 1.5, and 4.5 g kg^‐1^ day^‐1^, respectively) for 11 days. Weight trends were similar to the patterns from the earlier study (Table 1b). As shown in Figure 3, while there were no significant differences between the untreated and 0.5 g kg^‐1^ day^‐1^ patiromer‐treated groups, treatment with 1.5 and 4.5 g kg^‐1^ day^‐1^ patiromer significantly reduced serum K^+^ levels in hyperkalemic SHRs at day 11 (5.8 and 5.4 mmol/L, respectively, vs. 6.8 mmol/L in untreated animals, *p* < .01), to near levels observed in the control, uninduced SHR + UniNx animals. Mean levels of serum K^+^ were also significantly mildly elevated in the uninduced SHR + UniNx when compared with the WKY control animals at day 11 (4.9 mmol/L vs. 4.6 mmol/L, *p* < .01).

### 3.4 Effect of short‐term POC patiromer treatment and dose response on serum aldosterone

**FIGURE 4:**
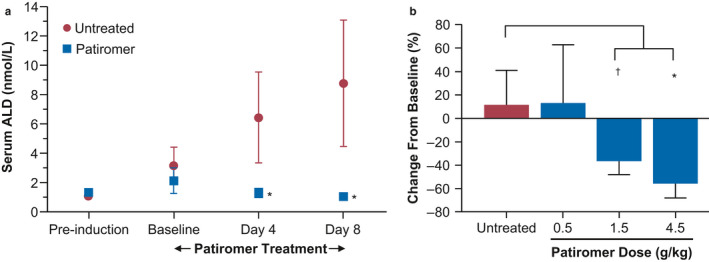
Patiromer effect on aldosterone (ALD) in the spontaneously hypertensive rats with hyperkalemia (SHR‐HK) model (short‐term proof‐of‐concept and dose‐response study). (a) SHRs were induced with HK and were either untreated (red circles) or treated with 4 g kg^‐1^ day^‐1^ patiromer (blue squares). (b) SHR‐HK were either untreated or treated with increasing doses of patiromer (0.5, 1.5, 4.5 g kg^‐1^ day^‐1^), and serum ALD was evaluated on day 11 of treatment. *N* = 8/group; data shown as mean ± standard deviation. Two‐way ANOVA followed by post hoc Bonferroni's multiple comparison posttest was used to compare untreated versus treated. *p < .0001; ^†^ *p* < .01

The effect of patiromer on lowering serum aldosterone along with serum K^+^ has been observed in human clinical trials (Weir et al., 2016); therefore, similar effects should be expected in an HK animal disease model. As shown in Figure 4a, short‐term treatment with patiromer (4 g kg^‐1^ day^‐1^) following HK induction in SHR‐HK resulted in significant reductions in serum aldosterone to baseline levels and was observed throughout the duration of the treatment period, starting at day 4 and continuing through day 8. The untreated animals experienced steady increases in serum aldosterone from baseline through day 8 as a result of HK induction (*p* < .0001 vs. untreated). As shown with serum K^+^ in Figure 3, the effect of patiromer was dose dependent in the mid‐ and high‐dose groups (36.5% and 55.6% reduction in 1.5 g kg^‐1^ day^‐1^ and 4.5 g kg^‐1^ day^‐1^ patiromer groups, respectively, *p* < .01) (Figure 4b *;* see Table 2 for actual aldosterone levels).

**TABLE 2:** Actual aldosterone levels for the dose‐response study from Figure 4b

Group	Baseline		Day 11		Difference (%)	*p* value
	Mean aldosterone (nmol/L)	*SD*	Mean aldosterone (nmol/L)	*SD*		
Untreated	3.76	0.77	4.05	0.97	7.73	NS
0.5 g/kg patiromer	3.70	0.75	4.27	2.67	15.30	NS
1.5 g/kg patiromer	3.89	0.68	2.47	0.68	−36.42	<.001
4.5 g/kg patiromer	3.85	1.00	1.62	0.24	−57.91	<.001

Note

NS, not significant.

### 3.5 Characterization of chronic HK induction and treatment with patiromer

**FIGURE 5:**
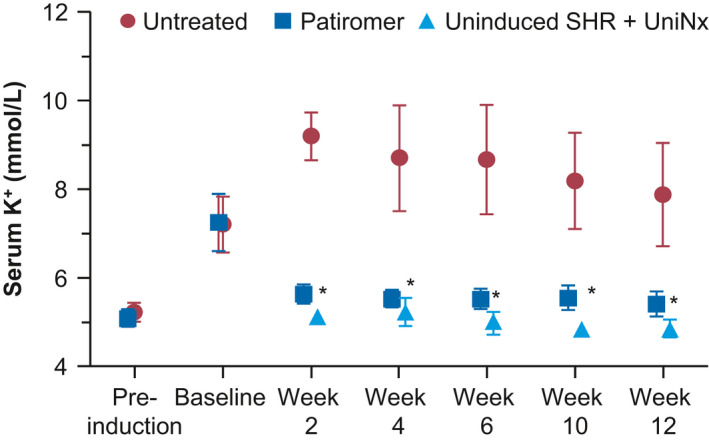
Effect on serum K^+^ with chronic patiromer treatment in the spontaneously hypertensive rats with hyperkalemia (SHR‐HK) model. SHRs were induced with HK chronically for 12 weeks. At baseline, SHR‐HK were either untreated (red circles) or treated with patiromer 4 g kg^‐1^ day^‐1^ (blue squares). Uninduced SHR + UniNx control (light blue triangles) were on a low K^+^ diet. *N* = 10/group; data shown as mean ± standard deviation. Two‐way ANOVA followed by post hoc Bonferroni's multiple comparison posttest was used to compare untreated versus treated. *p < .0001

Because we were able to successfully establish a hyperkalemic condition in SHRs that was tolerable in short‐term studies, we wanted to next examine the feasibility of long‐term, chronic induction in this animal model and to study its effects. HK was chronically induced for up to 12 weeks with the procedures mentioned in materials and methods. After achieving the HK state at day 5, SHRs were either untreated or treated with 4 g kg^‐1^ day^‐1^ of patiromer for a period of 12 weeks. As shown in Figure 5, chronic induction of HK resulted in an elevation of mean serum K^+^ from ~7 mmol/L at baseline to a peak of ~9 mmol/L at week 2 in the untreated SHR group. As the chronic induction continued through to week 12, mean serum K^+^ levels started to display a plateau effect and decreased gradually by ~1 mmol/L to final mean serum K^+^ of ~8 mmol/L by week 12. Interestingly, similar to the short‐term treatment, there were few or no signs of any significant clinical distress at elevated serum K^+^ levels. All animal groups experienced steady increases in weight from baseline through week 12 (Table 1c). In addition, serum creatinine values were significantly elevated from baseline through week 12, although there were no apparent significant differences between the groups (Table 3). This indicated that we were able to successfully induce and chronically maintain the hyperkalemic state along with CKD progression in SHRs without any significant adverse effects for a period of 12 weeks. Treatment with patiromer reduced mean serum K^+^ levels to near pre‐induction levels (*p* < .0001 vs. untreated group) and to those levels observed in untreated and uninduced SHR + UniNx control animals (on a low K^+^ diet) (Figure 5), indicating that the clinical responsiveness to a K^+^ binder in the SHR‐HK observed in the short‐term study was maintained in the chronic setting as well. Additionally, the effect of patiromer on serum K^+^ occurred almost completely within the first 2 weeks.

**TABLE 3:** Serum creatinine data for long‐term treatment study

Group	Baseline		Week 12	
	Mean serum creatinine (umol/L)	*SD*	Mean serum creatinine (umol/L)	*SD*
Untreated	45.09	4.42	52.17	4.42
Patiromer	47.75	3.54	52.17	6.19
Uninduced SHR + UniNx	36.25	3.54	56.59	7.07

Note

*p* < .05, week 12 versus baseline for all groups.

**FIGURE 6:**
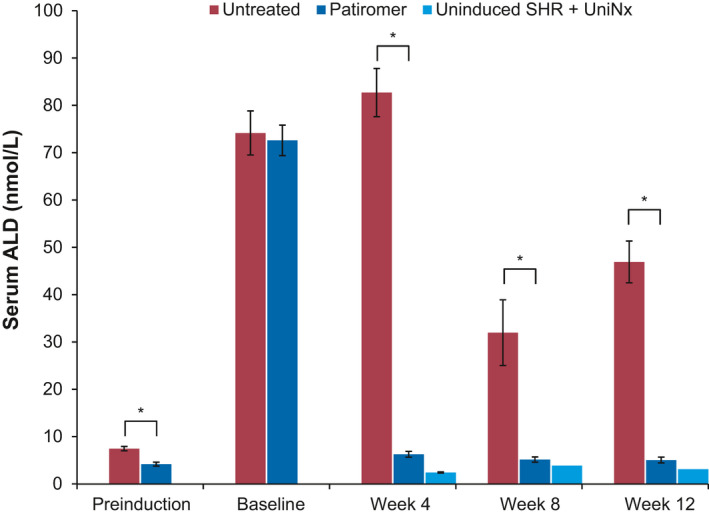
Effect on serum aldosterone (ALD) with chronic patiromer treatment in the spontaneously hypertensive rats with hyperkalemia (SHR‐HK) model. SHRs were induced with HK chronically for 12 weeks. At baseline, SHR‐HK were either untreated (red bars) or treated chronically with patiromer 4 g kg^‐1^ day^‐1^(dark blue bars). Uninduced SHR + UniNx controls (light blue bars) were on a low K^+^ diet. *N* = 10/group, data shown as mean ± standard deviation. The student's *t* test was used to compare untreated versus treated. *p < .001

Similarly, the effects on serum aldosterone were consistent with those observed in the short‐term study. As shown in Figure 6, significant elevations in serum aldosterone were observed from pre‐induction to baseline upon HK induction in both untreated and patiromer‐treated groups. Increases in serum aldosterone levels were much higher than those in the short‐term study due to a change in assay‐kit lots. While there appeared to be a statistically significant difference in aldosterone levels between the untreated and treated groups at pre‐induction (mean of 7.5 nmol/L in the untreated group vs. 4.2 nmol/L in the treated group, *p* < .001), both groups experienced a ~10–17‐fold rise in serum aldosterone to similar levels at the baseline time point (74.2 nmol/L in the untreated group vs. 72.6 nmol/L in the treated group, *p*  = NS). Similar to the rise in serum K^+^, the increase in aldosterone in the untreated group tended to peak early in the treatment duration and then exhibited a plateau effect toward the latter end of the study. Treatment with patiromer resulted in a dramatic lowering of serum aldosterone to near levels observed at pre‐induction and close to levels observed in uninduced SHR + UniNx control animals. This decrease was consistent across all time points (82.7 nmol/L in the untreated group vs. 6.3 nmol/L in the treated group at week 4; 32.0 nmol/L in the untreated group vs. 5.2 nmol/L in the treated group at week 8; 46.9 nmol/L in the untreated group vs. 5.1 nmol/L in the treated group at week 12; *p* < .001 between treated and untreated groups at all time points).

## 4 DISCUSSION

HK has been shown to be a chronically occurring condition, and the risk of elevated serum K^+^ is high in patients with CKD, DM, and/or HF (Albert et al., 2009; Einhorn et al., 2009; Yildirim et al., 2012). Clinical trials conducted with the recently available novel K^+^ binders have demonstrated that withdrawal of binders after achieving normokalemic serum ranges resulted in a recurrence of HK, indicating that the underlying pathological conditions that predispose patients to elevated serum K^+^ levels remain unchanged, warranting a continuous treatment approach (Packham et al., 2015; Weir et al., 2015). The availability of a chronic disease state model would significantly advance the field of study of this electrolyte imbalance.

Previously available HK models have been limited, with many studies typically utilizing 5/6 Nx approaches with additional dietary, pharmaceutical, or chemical intervention (Layton et al., 2018; Li et al., 2016). Dietary supplementation consisted of K^+^ in the form of potassium chloride in very high concentrations (upward of 15% for up to 2 weeks) to raise systemic potassium to hyperkalemic levels (DuBose & Good, 1992). Potassium‐sparing diuretics such as amiloride or spironolactone and antihypertensives such as angiotensin‐receptor blockers can cause HK, but either the study duration was short or the effect was minimal (Borok et al., 1987; Ma et al., 2014). Chemicals such as doxorubicin and adenine have also been used to induce CKD in rats, but alone may not induce HK and can increase mortality (Diwan et al., 2013, 2018; Li et al., 2016; Wapstra et al., 1999). We have also previously used a 5/6 Nx model with doxorubicin, trimethoprim, and quinapril supplementation to induce CKD and HK in rats. Although the animals were hyperkalemic over the 2‐week study period (Li et al., 2016), the treatment to induce HK was harsh on the animals. Viability and mortality of the rat and mouse models post‐HK induction (Askari et al., 2016; Li et al., 2016) have limited the utility of these models to study long‐term administration of therapeutic interventions such as novel K^+^ binders like patiromer (Bakris et al., 2015).

In this report, we developed a disease model that demonstrated a sustained ability to achieve and maintain HK chronically for up to 12 weeks. This condition could possibly be maintained even longer, though that has yet to be explored. Although the K^+^ levels remained quite elevated throughout the 12 weeks, we acknowledge that there was a downward trend following peak levels after the second week and it is possible that the HK will continue to attenuate over the next few months. Conceptually, our induction regimen had similar surgical, dietary, and pharmacological components as mentioned above, albeit with some key differences, including the animal strain selected as the pathological template. First, in our choice of animal strain, we deemed the SHR to be an appropriate starting point for developing a chronic hyperkalemic disease state because of several characteristics within the animal that are representative of the patient population at highest risk for HK (i.e., HTN, renal impairment). Moreover, the SHR model is a well‐established disease model and follows similar progression as human HTN, with pre‐, developing, and sustained periods of hypertensive phases (Doggrell & Brown, 1998). One very important feature of SHRs is that renal K^+^ handling is inherently compromised in this rat strain—possibly due to a genetic defect in the K^+^ transport in the distal nephron (Kau, Pritchard, & Leszczynska, 1992). We also chose the SHR model for the propensity of the animals to develop several renal defects that mimicked the pathological process of CKD, which is a key underlying comorbidity in patients who develop HK, in addition to those mentioned above. These include development of significant glomerular damage and proteinuria (beginning at ~6 weeks of age in males and rising steadily linearly from 10 to 70 weeks of age), vascular remodeling and hypertrophy, and reduction in glomerular filtration rate, specifically in male SHRs (Hultström, 2012; Mohamed & Sullivan, 2020; Yang, Zuo, & Fogo, 2010). Interstitial fibrosis and glomerulosclerosis develop at advanced age in SHRs, starting between 40 and 50 weeks (Yang et al., 2010). Furthermore, recent evidence has shown an impaired renal recovery following ischemia‐reperfusion injury in male SHRs results in exaggerated progression toward CKD when compared with females (Mohamed & Sullivan, 2020). Compared with other more traumatic manipulations such as the 5/6 Nx, some sequalae associated with CKD such as proteinuria and renal damage tend to progress more slowly in SHRs (Yang et al., 2010). Hypertensive kidney damage in this model is not apparent until ~30 weeks and significant renal failure occurs quite late at ~60 weeks of age (Hultström, 2012). Since the SHRs we used were young in their lifespan (7–9 weeks old), we accelerated time to renal damage and failure by performing UniNx (one kidney removed), and were able to do so, without seemingly compromising the animal's overall health. Indeed, we also observed a sustained increase in serum creatinine levels in our SHR‐HK and SHR‐UniNx animals as they aged, indicating that there was progressive renal degeneration that was in process. Thus, using SHRs with a UniNx and minimal drug and dietary intervention to induce chronic HK, this model may mimic disease sequelae in a significant segment of the CKD population (e.g., HTN, CKD, and HK), and may be considered as a valid surrogate CKD model to study chronic HK. The availability of one intact kidney also provided for a gentler and less traumatic dysfunctional renal status, enabling altered animals to recover better after surgery. Dietary intervention of a high K^+^ load was delivered to the animals by feeding them a high 3% (weight/weight) diet. The K^+^ salts used were a mixture of potassium chloride, dipotassium phosphite, potassium citrate monohydrate, and potassium carbonate. Finally, the pharmacological component of the induction regimen was kept minimal through the use of amiloride, a K^+^‐sparing diuretic that decreases renal K^+^ excretion to further drive elevation of serum K^+^. Despite the persistent HK, general observations and body‐weight measurements showed that the study animals remained healthy and did not present with any major adverse clinical signs and symptoms of distress.

In our animal studies, pre‐induction serum K^+^ levels ranged from 5–6 mmol/L in SHRs with UniNx. The pattern of induction revealed an early peak for serum K^+^ and aldosterone within the first 2 to 3 weeks to levels as high as ~9 mmol/L for mean serum K^+^, and then slight gradual reductions by ~1 mmol/L to final levels of ~8 mmol/L by the end of the study, when a steady state was finally achieved. Regression analysis confirmed a strong correlation between the levels of mean serum K^+^ and aldosterone. As mentioned above, this likely reflected the animals’ ability to adjust to high K^+^ levels with one intact kidney and thus to be more tolerant to the procedure, rather than the more traumatic 5/6 Nx (Okamoto & Aoki, 1963).

One of the pharmacodynamic (PD) parameters that is of interest is the effect of patiromer on serum aldosterone levels (Weir et al., 2016). In the patiromer OPAL‐HK phase 3 and AMETHYST‐DN phase 2 trials, along with reductions in serum K^+^, significant reductions in serum aldosterone levels were observed, along with concomitant reductions in blood pressure (Baba, 1986; Weir et al., 2015). Lowering serum aldosterone levels with patiromer could have important implications in patients with HK and CKD and/or HF. Although the inter‐relationship between plasma K^+^ concentration and aldosterone has been widely studied (Williams, 2005), the mechanism of the observed PD effects of chronic patiromer administration on serum aldosterone and blood pressure still remained unexplained, partly due to the limited availability of appropriate chronic disease animal models required to characterize the pharmacodynamics of K^+^‐lowering agents such as patiromer. The ability to chronically induce HK with minimal adverse consequences to the experimental animals provided the opportunity to examine responsiveness and validate the hyperkalemic pathological state in SHRs with the abovementioned PD effects of patiromer that were clinically observed. Indeed, our results indicate that this model is highly responsive to treatment with the K^+^ binder patiromer. Treatment with patiromer resulted in lowering of not only serum K^+^, but also serum aldosterone in the short‐term and long‐term chronic dosing studies. In humans, the maximum clinically available dose of patiromer is 25.2 g (Veltassa, 2015), which gives a drug:weight ratio of ~0.36 g/kg if dosed in a human with an average weight of ~70 kg. Therefore, the dose of 4 g/kg given in the rat is ~10–11 times higher than the highest clinically available dose to achieve the K^+^ reductions to near‐baseline levels. However, we also demonstrate that the effect of patiromer was dose dependent. Thus, these results are highly consistent with those observed in the patiromer clinical trials (Bakris et al., 2015; Weir et al., 2015, 2016) and now allow for further mechanistic exploration of other clinically important PD effects, such as the effect on systolic and diastolic blood pressure from the OPAL‐HK and AMETHYST‐DN studies (Bakris et al., 2015; Weir et al., 2016). Furthermore, the effects of elevated aldosterone and any long‐term effects on end‐organ damage such as fibrosis can also be investigated in this disease model.

Our studies have some limitations. In our animal studies, we have generally observed higher serum K^+^ levels in rats compared to those in humans, both at baseline and after HK induction. Even with high serum K^+^ levels at 8–9 mmol/L, surprisingly the animals were in good health. However, given that patients with end‐stage renal disease on hemodialysis can experience high K^+^ levels without undergoing typical symptoms, it is possible that this phenotype may not be that much different than humans. Next, the multiple interventions employed to bring about the high K^+^ levels may be construed as possibly too complex. However, we did observe that just surgical intervention through the UniNx only resulted in mild yet significantly elevated K^+^ levels (throughout the duration of the study; data not shown) when compared with WKY control animals (Figure 3, uninduced SHR + UinNx), and adding a 3% K^+^ diet did not lead to a higher serum K^+^ in these animals. These indicated that simply nephrectomizing the animals were only successful in incremental serum K^+^ increases, and that the combination of diet and drug interventions were required for markedly elevating K^+^ to the high levels that our model was able to achieve.

Another potential confounder in this model is the use of amiloride as the drug intervention to drive the K^+^ level elevation. Since it was orally administered in the drinking water at a fixed concentration in some of our studies, it is possible that the actual dose delivered may have animal‐to‐animal variability, depending on how much water was consumed. However, we measured the volume of amiloride containing water consumed per animal‐to‐estimate the daily average amiloride dose and found very minimal variation (data not shown). In addition, the K^+^ binder patiromer has also been shown to bind to certain medications when coadministered orally, with guidance provided in product labels to separate any orally concomitantly administered medication by a 3‐hr gap in order to prevent any unintended binding by the polymer (Lesko et al., 2017; Veltassa, 2015). In those reports, the possibility of patiromer binding to amiloride and inhibiting its action was not studied. Thus, it is possible that patiromer may bind to amiloride and interfere with its gastrointestinal absorption, which may contribute to the K^+^‐lowering effect observed with patiromer treatment of the SHR‐HK animals. We think this is unlikely, however, for several reasons. First, from our dose‐response study, the doses of 0.5, 1.5, and 4.5 g/kg patiromer in rats amount to ~1.4 times, ~4.1 times, and ~12.5 times the maximum clinically available dose of 25.2 g for a human subject of an average weight of ~70 kg (25.2 g/70 kg = 0.36 g/kg). If there were a potential interaction, we should not have observed a dose response, since all of the doses used were well above the ratio for the maximum clinically available dose, and thus should have seen a saturating response (i.e., same level of aldosterone reduction for all doses). Furthermore, as per the amiloride prescribing information (PI), it is to be dosed with food (Amiloride, 2018; Prescribers’ Digital Reference, 2020). Its absorption is ~50% and this is decreased by ~50% when given with food. Because amiloride was administered throughout the day in the drinking water (administered to the animals ad libitum) and not necessarily with or without food, absorption should be closer to the maximum for a rat (assuming absorption in rats is similar to humans). However, if there was, indeed, any binding of amiloride by patiromer, the amount of binding would need to exceed the effect of food on amiloride absorption before it would be clinically relevant as amiloride is recommended to be given with food per the PI. Regardless, without direct binding studies between patiromer and amiloride, we cannot preclude a possible interaction between the two agents, and thus acknowledge this as a potential limitation.

Finally, patiromer was the only K^+^ binder that was tested in this model. Other binders may be tested in future studies to determine if similar PD effects are present.

## 5 CONCLUSIONS

We have developed a reproducible animal model of chronic HK in SHRs. Patiromer significantly reduced serum K^+^ and aldosterone in hyperkalemic rats, and changes of K^+^ and aldosterone were well correlated. This novel animal disease model may be suitable for evaluating the effect of K^+^‐lowering therapies on K^+^‐mediated regulation of aldosterone and effects of the hormone.

## DISCLOSURES

S.P.N. Iyer and L. Li report employment by Relypsa, Inc., a Vifor Pharma Group Company, and stock in Vifor Pharma. L. Lee reports previous employment by Relypsa, Inc., a Vifor Pharma Group Company, during the time of the study.

## AUTHOR CONTRIBUTIONS

L. Lee and L. Li conceived and designed research; S.P.N. Iyer and L. Lee performed experiments and prepared figures; S.P.N. Iyer, L. Lee, and L. Li analyzed data, interpreted results of experiments, and approved the final of the manuscript; and S.P.N. Iyer and L. Li drafted the manuscript. S.P.N. Iyer, L. Li, and L. Lee had full access to all the data in the study and take responsibility for the integrity of data and the accuracy of the data analysis.

## Data Availability

The data that support the findings of this study are available upon reasonable request from the corresponding author.
